# Early and mid-term outcome of patients with low-flow–low-gradient aortic stenosis treated with newer-generation transcatheter aortic valves

**DOI:** 10.3389/fcvm.2022.991729

**Published:** 2022-10-06

**Authors:** Chiara Fraccaro, Giuseppe Tarantini, Stefano Rosato, Giovanni Baglio, Fausto Biancari, Marco Barbanti, Corrado Tamburino, Francesco Bedogni, Marco Ranucci, Gian Paolo Ussia, Fulvia Seccareccia, Paola D'Errigo

**Affiliations:** ^1^Interventional Cardiology Unit, Department of Cardiac, Thoracic, Vascular Sciences and Public Health, University of Padua, Padua, Italy; ^2^Centro Nazionale per la Salute Globale, National Center for Global Health, Istituto Superiore di Sanità Italiana, Roma, Italy; ^3^Italian National Agency for Regional Healthcare Services, Rome, Italy; ^4^Clinica Montevergine, GVM Care & Research, Mercogliano, Italy; ^5^Heart and Lung Center, Helsinki University Hospital, University of Helsinki, Helsinki, Finland; ^6^Division of Cardiology, A.O.U. Policlinico “G. Rodolico—San Marco”, University of Catania, Catania, Italy; ^7^Interventional Cardiology Unit, IRCCS Policlinico San Donato, Milan, Italy; ^8^Department of Cardiothoracic and Vascular Anesthesia and ICU, IRCCS Policlinico San Donato, Milan, Italy; ^9^Department of Cardiovascular Sciences, Campus Bio-Medico University of Rome, Rome, Italy

**Keywords:** low-flow–low-gradient, aortic stenosis, transcatheter aortic valve replacement, valvular heart disease, left ventricular dysfunction

## Abstract

Patients with non-paradoxical low-flow–low-gradient (LFLG) aortic stenosis (AS) are at increased surgical risk, and thus, they may particularly benefit from transcatheter aortic valve replacement (TAVR). However, data on this issue are still limited and based on the results with older-generation transcatheter heart valves (THVs). The aim of this study was to investigate early and mid-term outcome of TAVR with newer-generation THVs in the setting of LFLG AS. Data for the present analysis were gathered from the OBSERVANT II dataset, a national Italian observational, prospective, multicenter cohort study that enrolled 2,989 consecutive AS patients who underwent TAVR at 30 Italian centers between December 2016 and September 2018, using newer-generation THVs. Overall, 420 patients with LVEF ≤50% and mean aortic gradient <40 mmHg were included in this analysis. The primary outcomes were 1-year all-cause mortality and a combined endpoint including all-cause mortality and hospital readmission due to congestive heart failure (CHF) at 1 year. A risk-adjusted analysis was performed to compare the outcome of LFLG AS patients treated with TAVR (*n* = 389) with those who underwent surgical aortic valve replacement (SAVR, *n* = 401) from the OBSERVANT I study. Patients with LFLG AS undergoing TAVR were old (mean age, 80.8 ± 6.7 years) and with increased operative risk (mean EuroSCORE II, 11.5 ± 10.2%). VARC-3 device success was 83.3% with 7.6% of moderate/severe paravalvular leak. Thirty-day mortality was 3.1%. One-year all-cause mortality was 17.4%, and the composite endpoint was 34.8%. Chronic obstructive pulmonary disease (HR 1.78) and EuroSCORE II (HR 1.02) were independent predictors of 1-year mortality, while diabetes (HR 1.53) and class NYHA IV (HR 2.38) were independent predictors of 1-year mortality or CHF. Compared with LFLG AS treated with SAVR, TAVR patients had a higher rate of major vascular complications and permanent pacemaker, while SAVR patients underwent more frequently to blood transfusion, cardiogenic shock, AKI, and MI. However, 30-day and 1-year outcomes were similar between groups. Patients with non-paradoxical LFLG AS treated by TAVR were older and with higher surgical risk compared with SAVR patients. Notwithstanding, TAVR was safe and effective with a similar outcome to SAVR at both early and mid-term.

## Introduction

Transcatheter aortic valve replacement (TAVR) is as effective and safe as surgical aortic valve replacement (SAVR), and it has become the first-choice therapy in increased risk patients with severe aortic stenosis (AS) as well as otherwise lower-risk elderly (1). LFLG AS is present in about 5% to 10% of patients with or without ischemic cardiomyopathy (2). Left ventricular impairment is reversible after valve replacement when due to afterload mismatch or partially reversible when cardiomyopathy or myocardial fibrosis subsided. The treatment of these patients is challenging, and SAVR entails an increased operative mortality ranging from 6 to 30% (3–9). TAVR seems to be promising in this setting because of its increasing procedural safety (short operative time and no need for extracorporeal circulation with consequent better myocardial protection), as well as hemodynamic performance of transcatheter devices (10). However, TAVR downsides remain, such as post-procedural paravalvular leak (PVL) and need for permanent pacemaker (PPM), that might be detrimental in the setting of an already impaired LVEF (11, 12). To date, evidence supporting the value of TAVR in this setting is lacking (13–16).

In the last decade, the Italian Ministry of Health promoted the monitoring throughout the country of data regarding the treatments and outcomes of all patients affected by severe AS, which were collected into two national datasets: OBSERVANT I and OBSERVANT II. The primary aim of this study was to assess, in the large national study OBSERVANT II, the early and mid-term outcome of TAVR performed with newer-generation THVs in the setting of LFLG AS. Moreover, the secondary aim was to compare the outcome of these patients with that of patients who underwent SAVR included in the OBSERVANT I study (17).

## Materials and methods

### Data source

Data for this analysis were gathered from the OBSERVANT II datasets. OBSERVANT II was a national observational, prospective, multicenter cohort study that enrolled 2,989 consecutive AS patients who underwent TAVR at 30 Italian centers of cardiology between December 2016 and September 2018 (18–20). Twenty-eight centers met the data quality criteria required by the study protocol, and their data were included in the present analysis. The study protocol was approved by local ethics committees, and the recruited patients gave their consent to participate in this study. Data on baseline characteristics, operative details, and adverse events occurred during the index hospitalization were prospectively collected in an electronic case report form. Data on adverse events occurred after hospital discharge were gathered by a linkage with the National Hospital Discharged Records database provided by the Italian Ministry of Health and other administrative databases available through a collaboration with the Italian National Program for Outcome Evaluation (PNE-AGENAS). Linking to these national registries guaranteed complete follow-up data on outcomes at 1-year follow-up. For the secondary analysis, aimed at comparing outcomes between TAVR and SAVR patients, the SAVR historical cohort was obtained from the OBSERVANT I dataset that enrolled 7,618 consecutive AS patients who underwent TAVR (1,911 patients) or SAVR (5,707 patients) at 93 Italian centers between December 2010 and June 2012 (21).

### Study population

**Figure 1:**
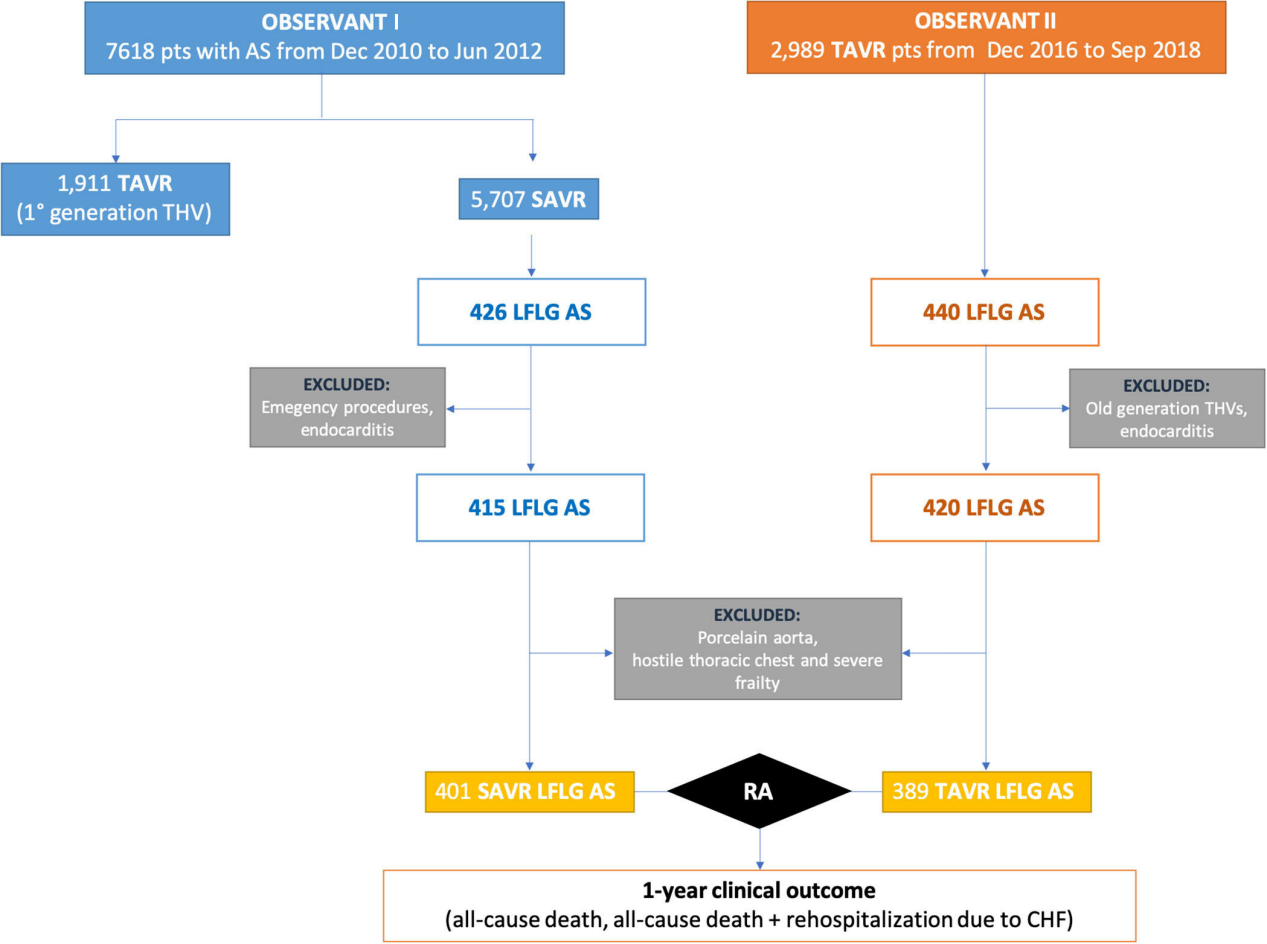
Study flowchart. AS, aortic stenosis; LFLG, low-flow–low-gradient; RA, risk adjustment; SAVR, surgical aortic valve replacement; TAVR, transcatheter aortic valve replacement.

Out of the 2,989 patients who underwent TAVR for severe AS and included in the OBSERVANT II dataset, 420 patients met the following inclusion criteria of this analysis: (1) left ventricular ejection fraction (LVEF) ≤50% and (2) mean aortic transvalvular gradient < 40 mmHg. Only those patients receiving new-generation THVs (Acurate, Boston Scientific, MA, USA; Evolut R and PRO, Medtronic, Minneapolis, MN, USA; Lotus, Boston Scientific, Marlborough, MA, USA; Portico, Abbott Vascular, Santa Clara, CA, USA; Sapien 3, Edwards Lifesciences Corp., Irvine, CA, USA; Engager, Medtronic 3F Therapeutics, Santa Ana, CA, USA) were included in this study. Active endocarditis was an exclusion criterion. Furthermore, LFLG TAVR patients (*n* = 389) from the OBSERVANT II dataset were compared with patients who underwent SAVR from the OBSERVANT I dataset (*n* = 401**)** (21). Active endocarditis, porcelain aorta, hostile chest, emergency procedure, and grade 3 of frailty based on the geriatric status scale (GSS) index (22) were exclusion criteria for this sub-analysis (Figure 1).

### Outcomes

The primary outcomes were 1-year all-cause mortality and the composite of mortality and hospital readmission due to congestive heart failure (CHF) at 1 year (this endpoint has been adopted as a surrogate of futility). The secondary outcomes were 30-day mortality and the following adverse events occurring during the index hospitalization: stroke, conversion to cardiac surgery, complication at the left ventricular apex, major vascular injury, acute kidney injury, post-operative change in estimated glomerular filtration rate (e-GFR), myocardial infarction (MI), permanent pacemaker (PPM) implantation, cardiogenic shock, infections, red blood cell transfusion, procedure for cardiac tamponade, as well as valve prosthesis performance defined as the mean and peak post-procedural transvalvular gradient and paravalvular regurgitation. Major vascular injury was defined as any vascular complication at peripheral access site requiring surgical or endovascular intervention. Infectious complications were defined as clinically proven surgical site infections, infections involving organs, and sepsis. Technical success, device success, and early safety were reported according to VARC-3 definitions (23). Moreover, the major adverse cardiac and cerebrovascular event (MACCE) was considered as a primary outcome of interest for the comparison between TAVR and SAVR groups. MACCE was defined as a composite of all-cause mortality, stroke, myocardial infarction, and/or coronary revascularization.

### Statistical analysis

Continuous variables were reported as means and standard deviations. Categorical variables were reported as counts and percentages. Missing data were not replaced. Differences between TAVR and SAVR groups were evaluated by the χ^2^ or Fisher exact test for categorical variables and by the *t*-test for continuous variables. Multivariate Cox proportional hazards regression models were used to identify independent predictors of 1-year mortality and 1-year composite endpoint of mortality and hospital readmission due to CHF in the TAVR population. Moreover, for the secondary aim multivariate Cox regression models were used to compare 1-year death, 1-year mortality + CHF, and 1-year MACCE between TAVR and SAVR patients. To validate the results obtained from this analysis, a propensity score approach with the inverse probability of treatment weighting (IPTW) method was used (Supplementary material). *P* < 0.05 was set for statistical significance. Statistical analyses were performed using SAS statistical software version 9.4 (SAS Institute, Cary, NC, USA).

## Results

### Baseline characteristics of LFLG TAVR

The mean age of TAVR patients was 81 years, and 65% were men. About one-third of them had diabetes and coronary artery disease. EuroSCORE II >4% was present in 84% of cases, and NYHA class ≥ III in 79.9% of them. The mean LVEF was 38%, and the mean transvalvular aortic gradient was 29 mmHg. A concomitant moderate-to-severe mitral regurgitation was observed in more than half of patients. Baseline clinical and echocardiographic characteristics are summarized in Table 1.

**Table 1:** Baseline characteristics of patients with LFLG AS included in OBSERVANT II dataset.

**Variables**	**Total cohort (*****n*** = **420)**	
	**Missing**	
Age, years	0	80.8 ± 6.7
Female sex	0	147 (35.0)
BMI, kg/m^2^	3	25.8 ± 4.4
Diabetes	1	135 (32.2)
Coronary artery disease	3	150 (36.0)
1 Vessel		74 (17.7)
2 Vessels		27 (6.5)
3 Vessels/LM		49 (11.8)
Previous myocardial infarction	2	
<90 days		13 (3.1)
>90 days		100 (23.8)
Previous PCI	1	95 (22.7)
Previous cardiac surgery	0	106 (25.2)
Previous CABG	0	81 (19.3)
Previous aortic surgery	1	19 (4.5)
Other prior cardiac surgery procedures	0	37 (8.8)
COPD	0	81 (19.3)
Oxygen therapy	3	16 (3.8)
e-GFR (ml/min/1.73 m^2^)	1	
≥45–60		108 (25.8)
≥30–45		109 (26.0)
≥15–30		30 (7.2)
<15		18 (4.3)
Dialysis	1	18 (4.3)
Neurological dysfunction	0	8 (1.9)
Porcelain aorta	5	22 (5.3)
Hostile chest	10	6 (1.5)
Peripheral artery disease	3	103 (24.7)
Liver disease	2	4 (1.0)
Pulmonary hypertension	0	34 (8.1)
Active cancer	2	16 (3.8)
EuroSCORE II, %	9	11.5 ± 10.2
EuroSCORE II >4 %	9	347 (84.4)
Frailty classes	0	
0		228 (54.3)
1		98 (23.3)
2		87 (20.7)
3		7 (1.7)
Critical preoperative status	0	16 (3.8)
NYHA classes	3	
I		2 (0.5)
II		82 (19.7)
III		293 (70.3)
IV		40 (9.6)
Unstable angina	0	29 (6.9)
Hemoglobin level, gr/dl	6	11.9 ± 1.7
Albumin level, gr/l	126	3.9 ± 0.7
AVA, cm^2^	21	0.7 ± 0.2
AV pick gradient, mmHg	18	50.2 ± 13.5
AV mean gradient, mmHg	0	29.6 ± 7.7
AV annulus diameter, mm	227	23.3 ± 2.5
Mitral regurgitation	1	
No/trivial		33 (8.5)
Mild		180 (46.6)
Moderate		162 (42.0)
Severe		44 (11.4)
LVEF, %	0	37.6 ± 8.6
30–50%		352 (83.8)
≤30%		68 (16.2)

Continuous variables are reported as mean and standard deviation. Categorical variables are reported as counts and percentages (in parentheses).

AV, aortic valve; AVA, aortic valve area; BMI, body mass index; CABG, coronary artery bypass grafting; COPD, chronic obstructive pulmonary disease; e-GFR, estimated glomerular filtration rate; LVEF, left ventricular ejection fraction; NYHA, New York Heart Association; PCI, percutaneous coronary intervention.

### Early outcome after TAVR for LFLG AS

**Figure 2:**
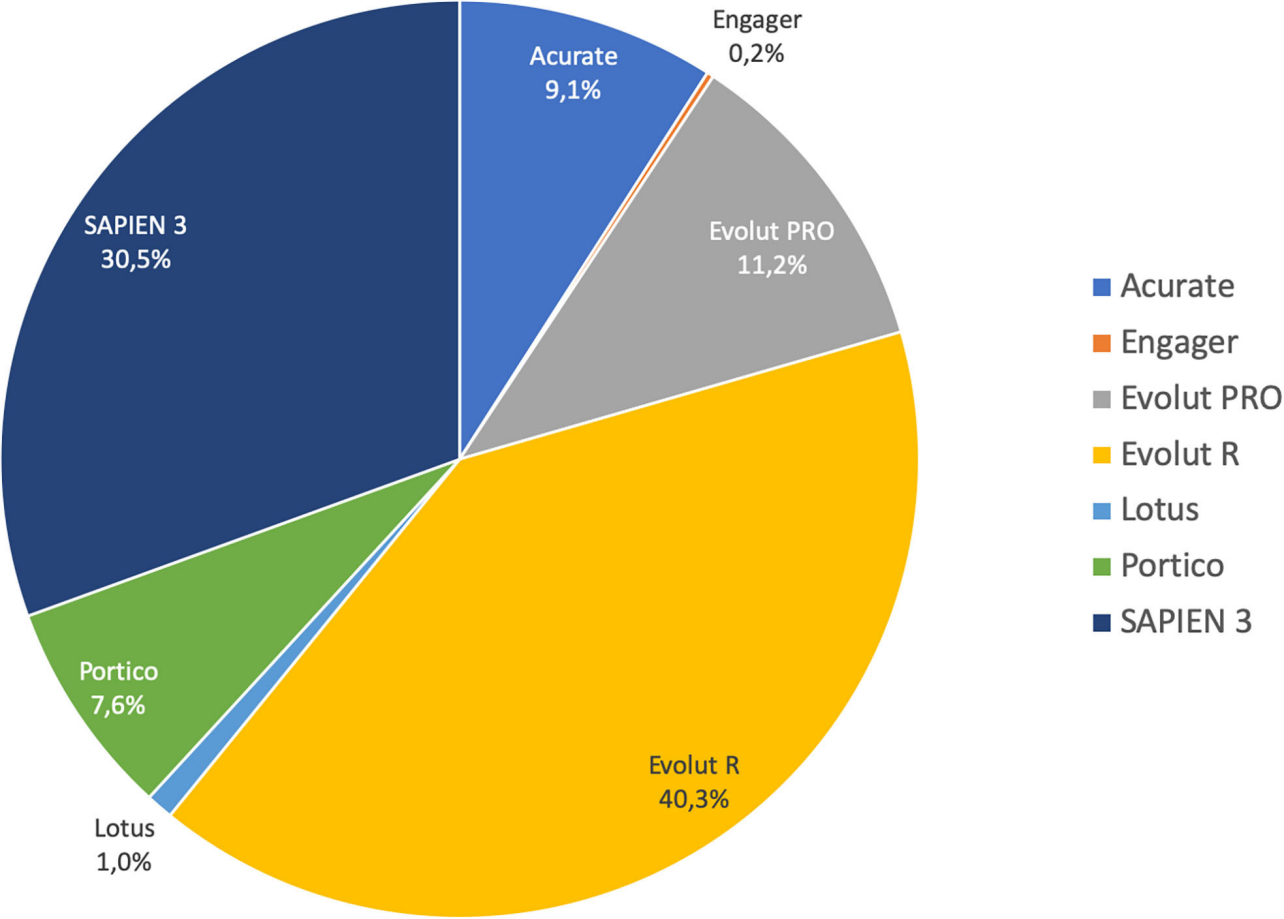
Pie chart showing the proportion of different devices implanted.

Most of the procedures were performed through a transfemoral approach (87%) using a self-expandable THV. The proportions of employed THVs are summarized in Figure 2. The rates of aortic valve pre- and post-dilatation were 31 and 18%, respectively. Mechanical circulatory support was needed in 1.4% of cases. Technical success was observed in 92% of cases. The device success rate at 30 days was 83% and early safety 68%. The mean transvalvular gradient significantly decreased from 29 mmHg to 8 mmHg (*p* < 0.001). Thirty-day mortality was 3.1%. Other early outcomes are summarized in Table 2.

**Table 2:** Early outcome of LFLG TAVR group.

**Variables**	**Total cohort (*****n*** = **420)**	
	**Missing**	
Technical success	0	386 (91.9)
Device success	0	350 (83.3)
Early safety	0	286 (68.1)
30-day mortality	0	13 (3.1)
Valve migration	2	7 (1.7)
Bailout TAVR-in-TAVR	0	8 (1.9)
Myocardial infarction	0	1 (0.2)
Major vascular complications	0	8 (1.9)
Complications at the apex	1	1 (0.2)
Permanent pacemaker	0	46 (11.0)
Moderate–severe PVL	0	32 (7.6)
Conversion to surgery	2	1 (0.2)
Stroke	0	8 (1.9)
Cardiogenic shock	2	8 (1.9)
Blood transfusion	2	46 (11.0)
AKI	1	11 (2.6)
Sepsis	0	2 (0.5)
Cardiac tamponade	0	4 (1.0)
Intensive care unit hospital stay (days)	32	1.4 ± 2.2

Continuous variables are reported as mean and standard deviation. Categorical variables are reported as counts and percentages (in parentheses).

AKI, acute kidney injury; PVL, paravalvular leak.

### One-year outcome after TAVR for LFLG AS

**Figure 3:**
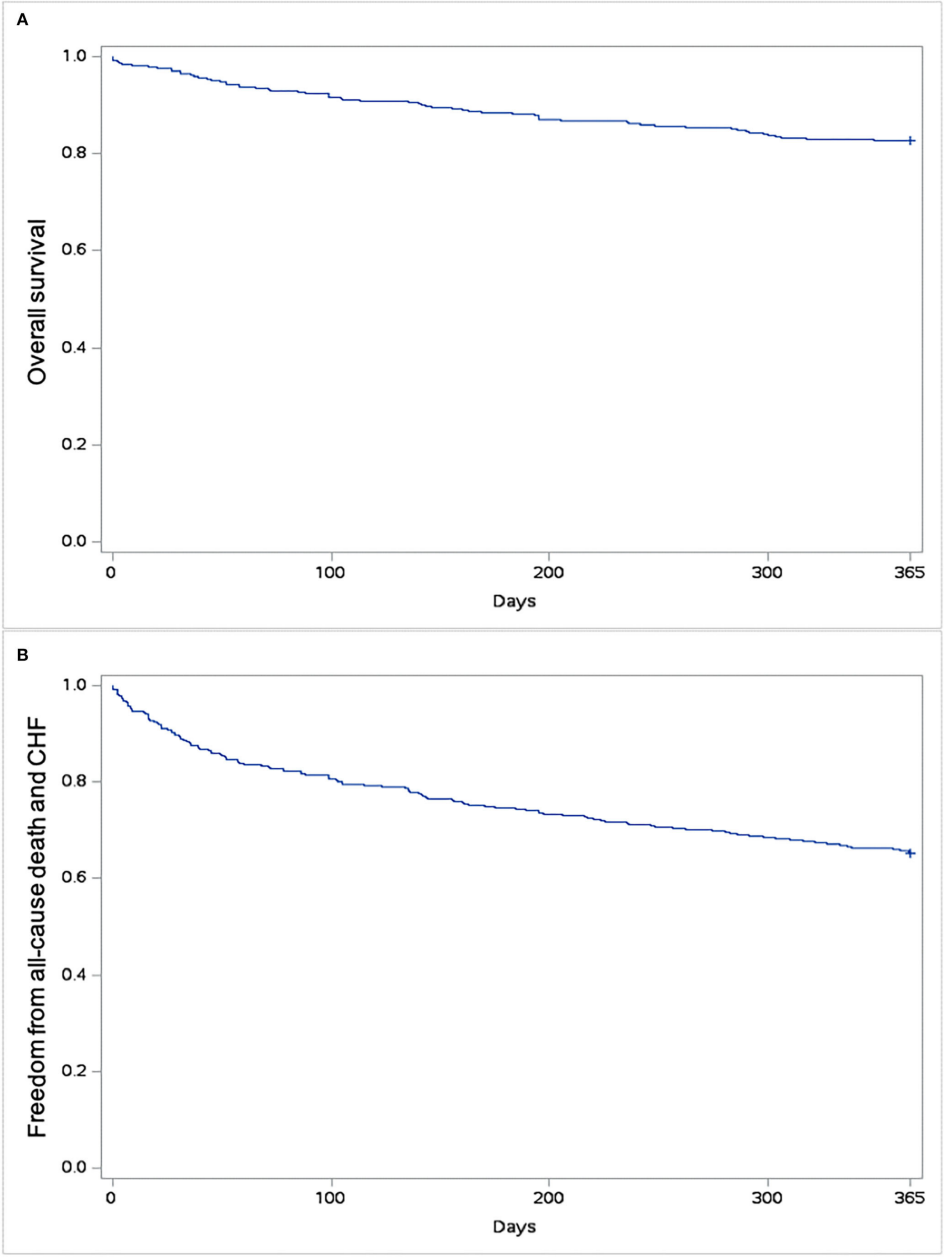
Kaplan–Meyer estimates of survival **(A)** and freedom from all-cause mortality and/or rehospitalization due to congestive heart failure (CHF) **(B)**.

One-year follow-up was complete in all patients. One-year all-cause mortality was 17.4%. The rate of hospital readmission due to CHF was 24.3%, and the rate of composite outcome was 34.8%. Kaplan–Meyer estimates of 1-year all-cause mortality and of combined all-cause mortality or CHF are shown in Figure 3. Predictors of 1-year mortality were chronic obstructive pulmonary disease and EuroSCORE II (Table 3). Predictors of combined 1-year composite outcome were diabetes and NYHA class IV before intervention (Table 4). The type of THV was not selected in the stepwise model for any of the considered outcomes, and even forcing that variable into the models, it did not reach any statistical significance.

**Table 3:** Independent predictors of 1-year mortality in LFLG AS treated with new-generation THV.

**Clinical variables**	**HR**	**P- value**	**CI 95%**	
Age, years	1.00	0.873	0.97	1.04
Female sex	0.68	0.167	0.40	1.17
Porcelain aorta	1.84	0.161	0.78	4.34
Hostile chest	2.75	0.126	0.75	10.01
COPD	1.78	0.036	1.04	3.05
Previous PCI	0.53	0.052	0.28	1.01
Concomitant PCI	0.50	0.178	0.19	1.37
e-GFR	0.99	0.122	0.98	1.00
EuroSCORE II	1.02	0.021	1.00	1.04
Mean aortic transvalvular gradient	0.98	0.189	0.95	1.01

COPD, chronic obstructive pulmonary disease; e-GFR, estimated glomerular filtration rate; PCI, percutaneous coronary intervention; THV, transcatheter heart valve.

**Table 4:** Independent predictors of combined 1-year mortality and rehospitalization due to congestive heart failure in LFLG AS treated with new-generation THV.

**Clinical variables**	**HR**	***p*-value**	**CI 95%**	
Age, years	1.00	0.763	0.98	1.03
Female sex	0.80	0.223	0.55	1.15
COPD	1.41	0.078	0.96	2.06
Neurological dysfunction	1.73	0.286	0.63	4.70
Previous aortic surgery	1.64	0.137	0.86	3.15
Diabetes	1.53	0.013	1.09	2.15
NYHA classes I and II	Ref			
NYHA class III	0.92	0.685	0.60	1.40
NYHA class IV	2.38	0.002	1.36	4.15

CHF, congestive heart failure; COPD, chronic obstructive pulmonary disease; NYHA, New York Heart Association.

### Outcome after SAVR or TAVR for LFLG AS

**Figure 4:**
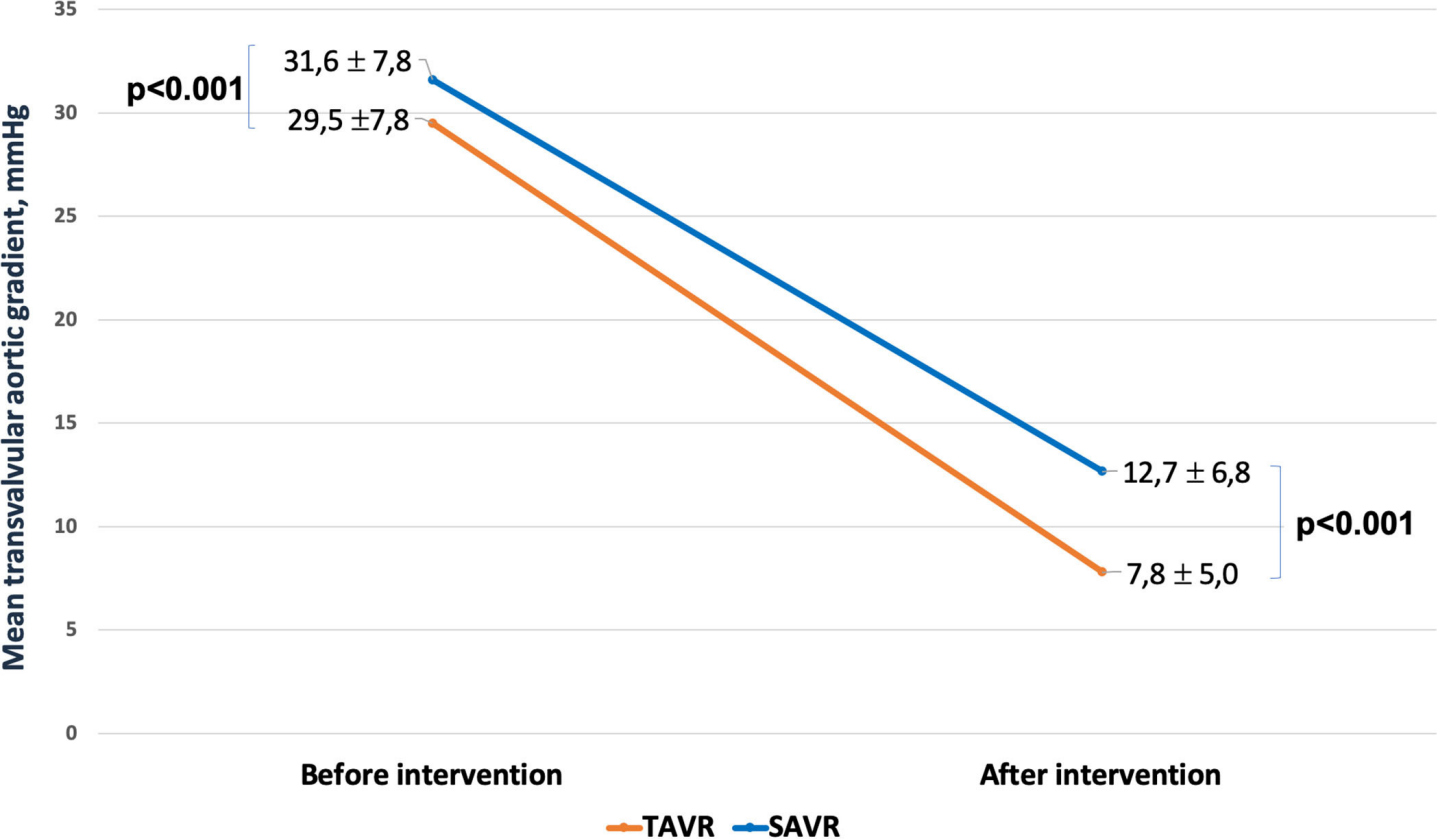
Change in mean transvalvular gradient after SAVR and TAVR.

**Figure 5:**
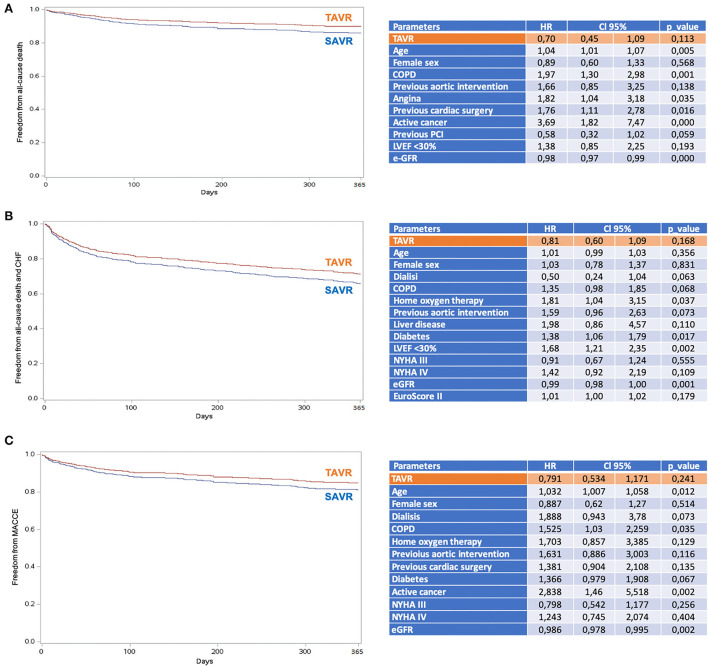
Adjusted proportional hazards estimates of outcomes after TAVR and SAVR. **(A)** Survival; **(B)** Survival freedom from the composite of mortality and hospital rehospitalization due to CHF; **(C)** Survival freedom from MACCE (all-cause mortality, stroke, myocardial infarction, and coronary revascularization). COPD, chronic obstructive pulmonary disease; e-GFR, estimated glomerular filtration rate; LVEF, left ventricular ejection fraction; NYHA, New York Heart Association; PCI, percutaneous coronary intervention; SAVR, surgical aortic valve replacement; TAVR, transcatheter aortic valve replacement.

Four-hundred and one patients underwent SAVR, and 389 patients underwent TAVR. SAVR patients were younger, less frail, and symptomatic, with a less impaired LV function which resulted in a lower EuroSCORE II (Supplementary Table 1). Still SAVR patients had a higher prevalence of CAD requiring concomitant CABG. Despite such differences, the unadjusted rates of 30-day mortality (SAVR, 4.5% TAVR, 3.3%, *p* = 0.407) and stroke (SAVR, 1.3%; TAVR 1.8%, *p* = 0.540) were similar between groups. However, SAVR patients required more frequently blood transfusion and suffered AKI, MI, and cardiogenic shock requiring longer intensive care unit stay. TAVR had a higher rate of major vascular complications, need for permanent pacemaker and residual PVL (Table 5). The reduction in post-procedural mean transvalvular gradient was lower in SAVR patients compared with TAVR patients (Figure 4). At 1 year, all-cause death was 14.0% after SAVR and 16.7% after TAVR (*p* = 0.284). About one-third of patients have died or required hospitalization for CHF, without significant difference between groups (33.2% after SAVR and 34.4% after TAVR; *p* = 0.704). An adjusted Cox proportional hazards analysis showed that survival, freedom from death or rehospitalization due to CHF, and freedom from MACCE were similar after SAVR and TAVR (Figure 5). The IPTW analysis (Supplementary Figures 1–5) confirmed the results observed with the adjusted Cox proportional hazards analysis.

**Table 5:** Early outcome of patients with LFLG AS treated with TAVR or SAVR.

**Outcomes**	**SAVR**, ***n*** = **401**		**TAVR**, ***n*** = **389**		***p*-value**
	**Missing**		**Missing**		
30-Day death	0	18 (4.5)	0	13 (3.3)	0.407
Valve migration	0	0 (0.0)	2	7 (1.8)	0.007
Myocardial infarction	3	7 (1.8)	0	1 (0.3)	0.036
Major vascular complications	25	1 (0.3)	0	8 (2.1)	0.022
Permanent pacemaker	4	18 (4.5)	0	44 (11.3)	<0.001
Stroke	5	5 (1.3)	0	7 (1.8)	0.540
Cardiogenic shock	5	35 (8.8)	2	8 (2.1)	<0.001
Blood transfusion	25	210 (55.9)	2	41 (10.6)	<0.001
Transfused RBC units	0	2.0 ± 3.1	0	0.2 ± 0.8	<0.001
AKI	11	38 (9.7)	1	11 (2.8)	<0.001
PCI	3	1 (0.3)	0	0 (0.0)	0.323
Tamponade	4		0		0.319
Requiring surgery		6 (1.5)		2 (0.5)	
Requiring percutaneous treatment		1 (0.3)		2 (0.5)	
Infectious complications	11	24 (6.2)	0	15 (3.9)	0.148
PVL	37		0		<0.001
No/trivial		326 (89.6)		217 (55.8)	
Mild		32 (8.8)		144 (37.0)	
Moderate		4 (1.1)		25 (6.4)	
Severe		2 (0.6)		3 (0.8)	
AV pick gradient, mmHg	91	23.1 ± 10.3	22	14.6 ± 8.6	<0.001
AV mean gradient, mmHg	95	12.7 ± 6.8	7	7.8 ± 5.0	<0.001
Intensive care unit stay, days	7	4.0 ± 6.7	7	1.4 ± 2.3	<0.001

Continuous variables are reported as mean and standard deviation. Categorical variables are reported as counts and percentages (in parentheses).

AKI, acute kidney injury; AV, aortic valve; PCI, percutaneous coronary intervention; PVL, paravalvular leak; RBC, red blood cells; SAVR, surgical aortic valve replacement; TAVR, transcatheter aortic valve replacement.

## Discussion

**Figure 6:**
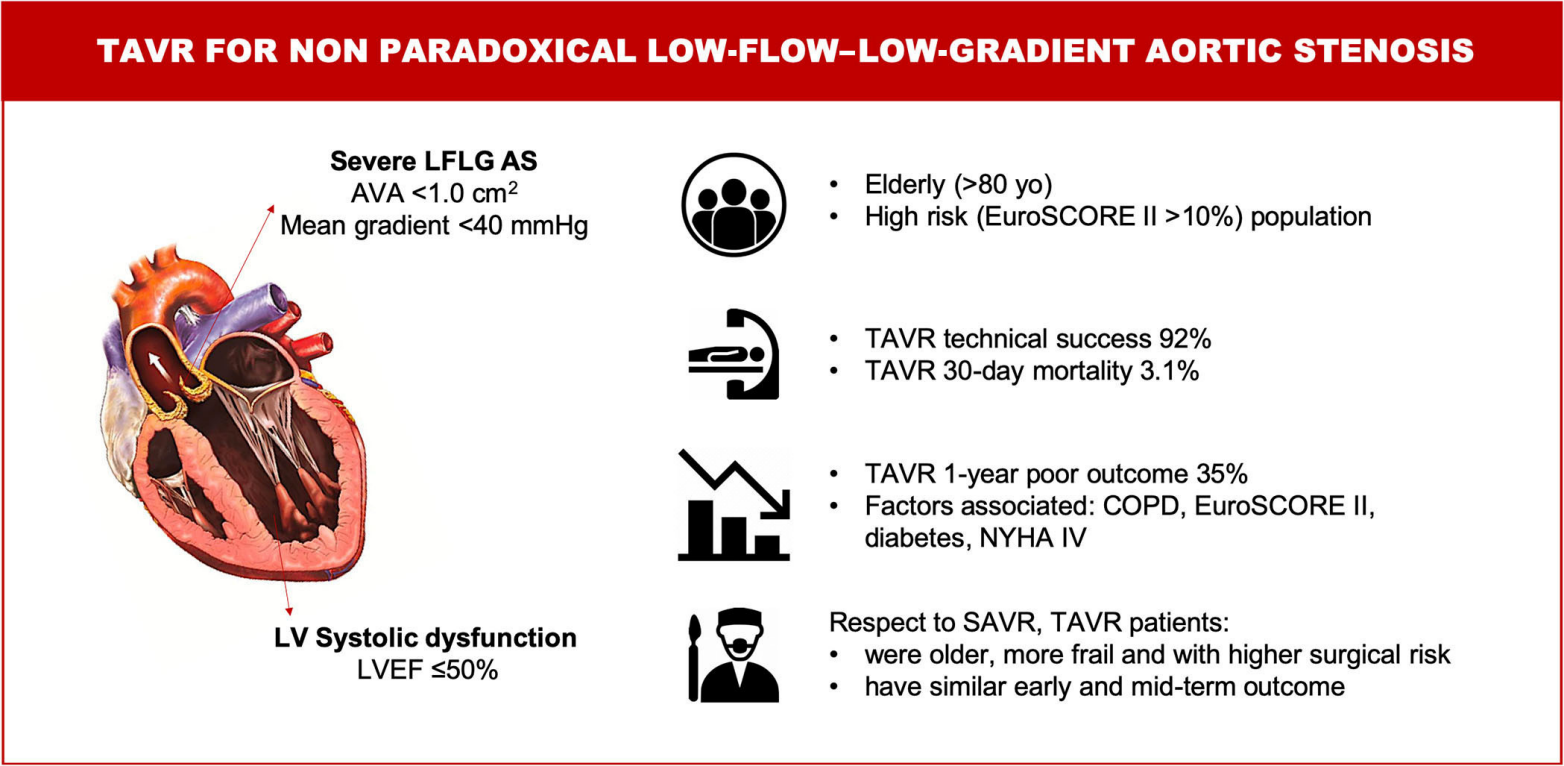
Central illustration. TAVR for non-paradoxical low-flow–low-gradient aortic stenosis. Characteristics, management and outcomes. AS, aortic stenosis; AVA, aortic valve area; COPD, chronic obstructive pulmonary disease; LV, left ventricular; LVEF, left ventricular ejection fraction; LFLG, low-flow – low-gradient; NYHA, New York Heart Association; SAVR, surgical aortic valve replacement; TAVR, transcatheter aortic valve replacement.

The main findings of this study including a large series of patients with non-paradoxical LFLG AS treated with the last-generation THVs can be summarized as follows: (1) TAVR appears to be a safe treatment strategy in this high-risk population, with a 30-day mortality rate lower than that expected by an estimated operative risk; (2) one-third of the patients experienced death and/or rehospitalization due to CHF during the first year; and (3) TAVR-treated patients were older, more frail, and with a higher surgical risk compared with SAVR-treated patients. Notwithstanding, TAVR was as safe and effective as SAVR at early and mid-term (Figure 6, Central Illustration).

To the best of our knowledge, this is the largest series evaluating the outcome of TAVR performed with modern THVs devices in patients with LFLG AS. The risk profile of our study population was similar to that of the 287 patients with LFLG AS undergoing TAVR with similar devices included in the TOPAS trial (16). In that registry, Ribeiro et al. observed a favorable early outcome of TAVR in their high-risk cohort, with mortality rates of 3.8 and 20.1% at 30 days and 1 year, respectively, which are similar to the results of this study (3.1 and 17.4%, respectively). The minimally invasive nature of TAVR that avoids the use of extracorporeal circulation and the risk of myocardial injury, the prevalent transfemoral approach, and better hemodynamic performance and low delivery profile of most recent THVs, as well as increasing experience, might have contributed to these results. Indeed, prior series including older-generation devices reported less favorable results. In fact, in the GARY registry (14), hospital mortality was 7.8% and 1-year mortality was 32.2% among LFLG AS patients treated with TAVR.

However, about one-third of our population still may die or have a recurrence of CHF within the first year after TAVR. These findings are similar to the figures observed in the TOPAS trial (16). Similarly, a poor outcome was observed in 33% of patients in the PARTNER trial (24), whereas the rate of death, stroke, or rehospitalization was 27% in the PARTNER 2A trial (25) and 17% in the SURTAVI trial (26). This raises the question of the need to assess the potential futility in patients undergoing TAVR. Clinical futility means the lack of clinical benefit within the first year after treatment, and it has been variably defined as the composite of death, rehospitalization due to CHF, or lack in functional recovery and improvement in quality of life (27). We observed that COPD and EuroSCORE II were independent predictors of 1-year mortality after TAVR and that diabetes and NYHA class IV symptoms independently predicted 1-year death and/or rehospitalization due to CHF. Low baseline mean gradient, anemia, renal failure, and the presence of moderate or severe post-procedural PVL after TAVR have been identified as other predictors of poor outcome in these patients (16, 28–30). However, a validated risk assessment tool for potential futility of TAVR is still lacking and the identification of patients who may not fully benefit from TAVR remains a dilemma for the heart team during the decision-making process.

When compared with an historical series of LFLG AS patients who underwent SAVR, TAVR patients included in the OBSERVANT II study were older, at a higher surgical risk, more fragile, with more comorbidities, and at an advanced stage of disease. Notwithstanding, 30-day mortality and stroke were similar between the two cohorts. Looking at post-procedural mean transvalvular gradient, THVs performed better than surgical aortic valve prostheses. This superiority of THVs is well-known, in particular with the use of supra-annular devices (60.8% in our series) (31–33). This is a point in favor of TAVR when discussing the best therapeutic option in the case of LFLG AS, as the more complete is the relief of afterload mismatch, the higher is the probability of LV recovery (34, 35). On the contrary, THVs still suffer from a higher rate of PVL and conduction disorders needing PPM implantation. The impact of PVL and PPM on clinical outcome and LV recovery after TAVR is still a matter of debate, and the scientific evidence on this field are controversial. Previous reports suggested that mild PVL is commonly observed after TAVR and usually leads to a benign outcome (36). However, a meta-analysis of 45 studies including around 13,000 patients concluded that moderate or greater post-TAVR PVL was associated with a more than 2-fold increase in overall all-cause mortality (37). Moreover, conduction disorders and PPM leading to cardiac dyssynchrony can be badly tolerated by an already impaired LVEF, as suggested by Weber et al. (11).

Beyond these differences, at risk-adjusted analysis for other confounding factors, we found that 1-year clinical outcome was similar between TAVR and SAVR patients. The same finding was reported by a sub-analysis of the PARTNER trial, where, after a small early hazard associated with SAVR in the first 30 days, both TAVR and SAVR similarly improved outcome with respect to medical therapy alone (13). These results have been further confirmed by a recent meta-analysis, showing that aortic valve replacement was associated with a significant decrease in all-cause mortality regardless of surgical or transcatheter approach and in all subclasses of LG AS (38).

There are some study limitations that deserve to be acknowledged. First, this is a prospective registry without an external event adjudication committee and echocardiographic data were site-reported without analysis in a centralized core laboratory. Second, no data were available on either Agatston calcium score or contractile reserve evaluated by low-dose dobutamine echocardiography. However, recent evidence suggest no impact of contractile reserve on outcome after TAVR (39). Third, frailty was estimated through a simple toolset (Geriatric Status Scale) including only basic daily life activities and cognitive impairment; however, other variables relative to nutritional status (BMI, albumin, hemoglobin) were collected separately. Data about futility are underreported with respect to VARC-3 definition (23), as we included only death and rehospitalization due to CHF, while data about quality of life at follow-up are lacking. Moreover, we do not know the reasons for death (cardiac or not cardiac) at follow-up. Additionally, TAVR and SAVR cohorts are from different time periods (2010–2012 for SAVR group and 2016–2018 for TAVR group), thus potentially reflecting different patient's selection and decision-making process by the local heart teams. However, as a matter of fact, no major changes in surgical techniques and technologies have been introduced during that time frame. Accordingly, we would not expect a significant variation in the results if a more contemporaneous surgical series had been available. Finally, both OBSERVANT I and OBSERVANT II are multicenter studies, and thus, the “center effect” should theoretically be considered. However, the small number of patients per center fulfilling inclusion criteria for this sub-analysis does not allow considering the variable “center” in the models, which would become highly unstable from the statistical point of view.

In conclusion, in patients with non-paradoxical LFLG AS, TAVR was as safe and effective as SAVR at early and mid-term intervals. TAVR was associated with a lower risk of severe early adverse events and therefore might be of benefit over SAVR in elderly with non-paradoxical LFLG AS. Attention should be paid to procedural planning to optimize hemodynamic acute result and reduce the risk of PVL and PPM. The risk of futility is impending, and predictors of poor outcome are still a matter of debate. Further studies are needed to address these issues and improve patients' selection.

## Data availability statement

The raw data supporting the conclusions of this article will be made available by the authors, without undue reservation.

## Ethics statement

The studies involving human participants were reviewed and approved by Segreteria del Comitato Etico per la Sperimentazione Clinica della Provincia di Padova, U.O.S.D. The patients/participants provided their written informed consent to participate in this study.

## Author contributions

CF, GT, and FS contributed to conception and design of the study. SR organized the database. PD'E performed the statistical analysis. CF wrote the first draft of the manuscript. GB, FBi, MB, CT, FBe, MR, and GU wrote sections of the manuscript. All authors contributed to manuscript revision, read, and approved the submitted version.

## Funding

The OBSERVANT study was supported by a grant (Fasc. 1M30) from the Italian Ministry of Health and Istituto Superiore di Sanità. The OBSERVANT II study was supported by the Italian Ministry of Health within the call Ricerca Finalizzata 2016 (code PE-2016-02364619).

## Conflict of interest

The authors declare that the research was conducted in the absence of any commercial or financial relationships that could be construed as a potential conflict of interest.

## Publisher's note

All claims expressed in this article are solely those of the authors and do not necessarily represent those of their affiliated organizations, or those of the publisher, the editors and the reviewers. Any product that may be evaluated in this article, or claim that may be made by its manufacturer, is not guaranteed or endorsed by the publisher.
