# Supplementary material for: Early and mid-term outcome of patients with low-flow–low-gradient aortic stenosis treated with newer-generation transcatheter aortic valves
Source: Front Cardiovasc Med. 2022 Oct 6;9:991729. doi: 10.3389/fcvm.2022.991729 (PMC9583539; doi:10.3389/fcvm.2022.991729)
Supplement: Supplementary file 1 [file Data_Sheet_1.docx]

**Supplemental material**

**Early and mid-term outcome of patients with low-flow – low-gradient aortic stenosis treated with newer-generation transcatheter aortic valves**

Fraccaro C et al.

**Supplemental Table 1. Baseline characteristics of patients with LFLG AS treated with TAVR or SAVR.**

| Clinical variables | SAVR  (N=401) | TAVR  (N=389) | P Value |
| --- | --- | --- | --- |
| Age, years | 73.5 ± 9.1 | 80.9 ± 6.5 | <0.001 |
| Female Sex | 127 (31.7) | 138 (35.5) | 0.258 |
| BMI | 26.8 ± 4.5 | 25.8 ± 4.4 | <0.001 |
| Diabetes | 128 (31.9) | 125 (32.2) | 0.929 |
| CAD  1-vessel  2-vessels  3-vessels/LM | 67 (16.7)  48 (12.0)  89 (22.2) | 64 (16.6)  26 (6.7)  44 (11.4) | <0.001 |
| Previous AMI  <90 days  >90 days | 39 (9.9)  57 (14.5) | 9 (2.3)  92 (23.7) | <0.001 |
| Previous PCI | 40 (10.0) | 88 (22.7) | <0.001 |
| Previous cardiac surgery | 25 (6.3) | 99 (25.4) | <0.001 |
| Previous CABG | 12 (3.0) | 76 (19.5) | <0.001 |
| Prior aortic surgery | 17 (4.3) | 16 (4.1) | 0.912 |
| Previous other cardiac surgery | 12 (3.0) | 34 (8.7) | <0.001 |
| COPD | 61 (15.2) | 77 (19.8) | 0.090 |
| Home oxygen therapy | 10 (2.5) | 15 (3.9) | 0.282 |
| e-GFR classification  ≥90  ≥60-90  ≥45-60  ≥30-45  ≥15-30  <15 | 54 (13.7)  156 (39.5)  102 (25.8)  51 (12.9)  16 (4.1)  16 (4.1) | 23 (5.9)  117 (30.2)  104 (26.8)  99 (25.5)  29 (7.5)  16 (4.1) | <0.001 |
| Dialysis | 14 (3.5) | 17 (4.4) | 0.520 |
| Neurological dysfunction | 10 (2.5) | 8 (2.1) | 0.665 |
| Peripheral vasculopathy | 77 (19.4) | 95 (24.6) | 0.078 |
| Liver disease | 10 (2.5) | 4 (1.0) | 0.115 |
| Pulmonary hypertension | 35 (9.5) | 32 (8.2) | 0.534 |
| Active cancer | 5 (1.3) | 15 (3.9) | 0.020 |
| EuroSCORE II | 6.0 ± 7.4 | 11.4 ± 9.8 | <0.001 |
| EuroSCORE II >4 | 172 (48.0) | 322 (84.3) | <0.001 |
| Frailty class  0  1  2 | 329 (82.0)  47 (11.7)  25 (6.2) | 217 (55.8)  92 (23.7)  80 (20.6) | <0.001 |
| Urgent status | 29 (7.2) | 32 (8.2) | 0.593 |
| NYHA functional class  I  II  III  IV | 23 (5.8)  150 (37.9)  173 (43.7)  50 (12.6) | 2 (0.5)  75 (19.4)  273 (70.5)  37 (9.6) | <0.001 |
| Unstable Angina | 38 (9.6) | 25 (6.4) | 0.100 |
| Hb level, gr/dl | 12.5 ± 1.7 | 11.9 ± 1.8 | <0.001 |
| Albumin level, gr/l | 3.7 ± 0.9 | 3.9 ± 0.7 | <0.001 |
| Need for concomitant coronary revascularization | 164 (41.9) | 30 (7.7) | <0.001 |
| Mitral regurgitation  no/trivial  mild  moderate  severe | 136 (33.9)  181 (45.1)  72 (18.0)  12 (3.0) | 31 (8.0)  167 (43.0)  150 (38.7)  40 (10.3) | <0.001 |
| LVEF <30% | 45 (11.2) | 64 (16.5) | 0.033 |
| LVEF, % | 40.3 ± 8.0 | 37.5 ± 8.6 | <0.001 |
| AVA, cmq | 0.8 ± 0.3 | 0.7 ± 0.3 | <0.001 |
| AV pick gradient, mmHg | 54.7 ± 15.2 | 50.0 ± 13.6 | <0.001 |
| AV mean gradient, mmHg | 31.6 ± 7.8 | 29.5 ± 7.8 | <0.001 |

Continuous variables are reported as mean and standard deviation. Categorical variables are reported as counts and percentages (in parentheses).

AV = aortic valve; AVA = aortic valve area; BMI = body mass index; CAD = coronary artery disease; CABG = coronary artery bypass graft; COPD = chronic obstructive pulmonary disease; GFR = glomerular filtration rate; LVEF = left ventricular ejection fraction; NYHA = New York Heart Association; PCI = percutaneous coronary intervention; SAVR = surgical aortic valve replacement; TAVR = transcatheter aortic valve replacement.

**Propensity score analysis**

A propensity score analysis using the inverse probability of treatment weighting (IPTW) method was used to adjust for differences in the baseline characteristics of patients who underwent SAVR or TAVR. A logistic regression model with a non-parsimonious approach was used to estimate the propensity score. Patients characteristics used to estimate the propensity score were the following: age, gender, EuroSCORE II, body mass index (BMI), dialysis, chronic obstructive pulmonary disease, neurological dysfunction, peripheral vasculopathy, home oxygen therapy, previous aortic intervention, critical pre-operative clinical status, unstable angina, liver disease, previous myocardial infarction, diabetes, active cancer, pulmonary hypertension, previous percutaneous coronary intervention, previous coronary artery bypass graft, left ventricle ejection fraction, frailty, NYHA functional class, coronary artery disease, mitral regurgitation, eGFR, urgency status, prior aortic surgery.

Being p the propensity score, a weight equal to 1 was used for patients treated with TAVI and equal to p/(1-p) for patients treated with SAVR. To address the issue of extreme weights, that often occurs when a treatment is not suitable for all members of the population, stabilized weights were used, truncating the weights to a defined threshold. A recursive method was used to select the best threshold of propensity score to be used. Observations with a p>0.75 were excluded.

**Supplemental Figure 1.** Standardized mean differences.


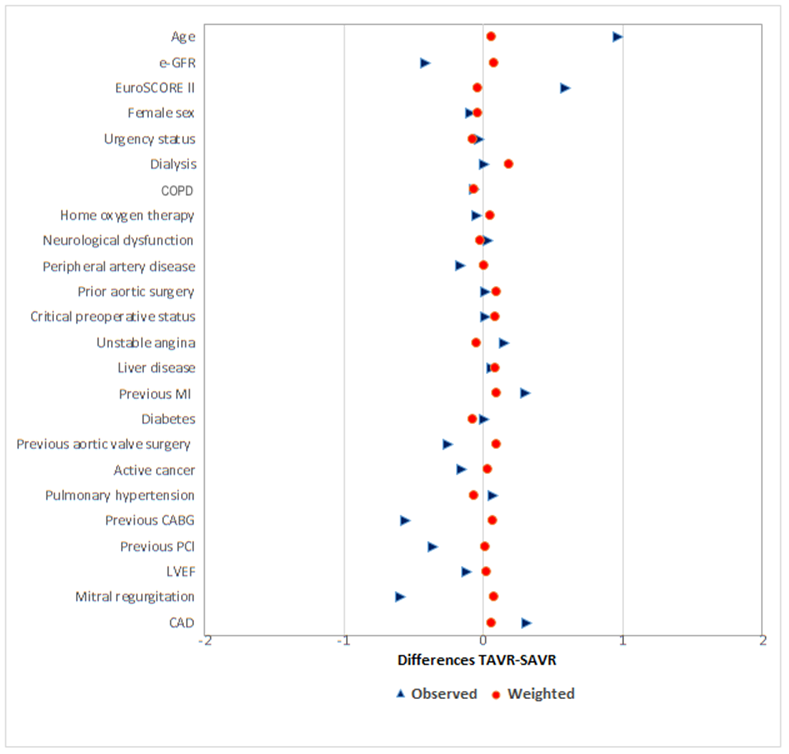


**Supplemental Figure 2. Log-PS distribution**

**
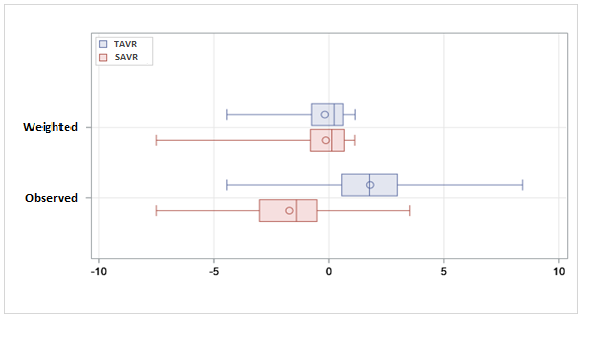
**

**Supplemental Figure 3.** Overall survival in the study cohorts.


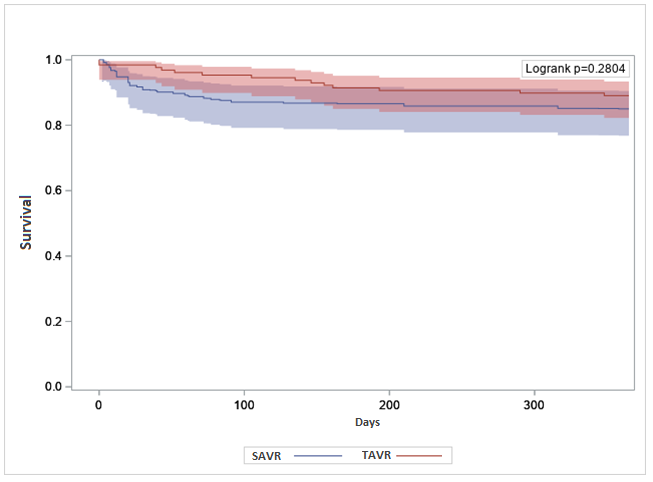


|  | Hazard ratio | p-value | 95%CI | |
| --- | --- | --- | --- | --- |
|  |  |  |  |  |
| TAVR | 0.69 | 0.295 | 0.350 | 1.375 |

**Supplemental Figure 4.** All cause death +CHF


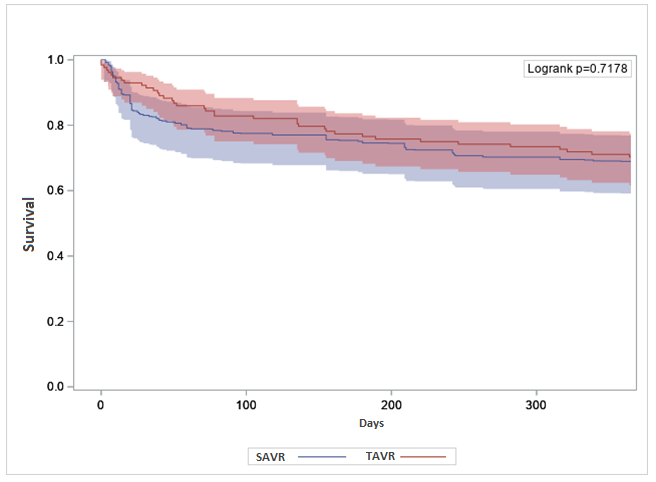


|  | Hazard ratio | p-value | 95%CI | |
| --- | --- | --- | --- | --- |
|  |  |  |  |  |
| TAVR | 0.92 | 0.698 | 0.590 | 1.424 |

**Supplemental Figure 5.** MACCE


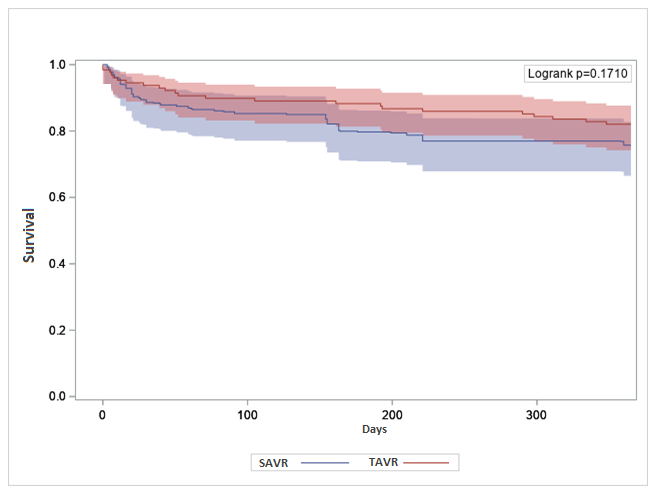


|  | Hazard ratio | p-value | 95%CI | |
| --- | --- | --- | --- | --- |
|  |  |  |  |  |
| TAVR | 0.71 | 0.207 | 0.415 | 1.209 |

**OBSERVANT II RESEARCH GROUP**

*Coordination*

Fulvia Seccareccia, Paola D'Errigo, Stefano Rosato, Gabriella Badoni.

National Centre for Global Health - Istituto Superiore di Sanità, Rome, Italy;

*Collaborators for the “Ricerca Finalizzata 2016” (PE-2016-02364619)*

Corrado Tamburino (PI), Davide Capodanno (Co-PI), Marco Barbanti. A.O.U. Policlinico “G. Rodolico – San Marco” – University of Catania, Catania, Italy

Fausto Biancari. Helsinki University Hospital and University of Helsinki, Helsinki, Finland; Oulu University Hospital, Oulu, Finland; Clinica Montevergine, GVM Care & Researchm, Mercogliano, Italy

Giovanni Baglio, Francesco Cerza. Agenzia Nazionale per i Servizi Sanitari Regionali (Age.Na.S) – PNE, Rome, Italy

Andrea Marcellusi. Faculty of Economics, University of Rome “Tor Vergata”, Rome, Italy

*Representatives of the Scientific Societies*

- IFC -  Italian Federation of Cardiology

Gennaro Santoro. Fondazione "G. Monasterio" CNR/Tuscany Region for the Medical Research and Public Health, Massa, Italy

Gian Paolo Ussia. Campus Bio-Medico University of Rome, Rome, Italy

- GISE – Italian Society of Interventional Cardiology

Giuseppe Musumeci. S. Croce e Carle Hospital, Cuneo

Francesco Bedogni. IRCCS Policlinico S. Donato, S. Donato Milanese, Milan, Italy

Sergio Berti. Fondazione "G. Monasterio" CNR/Tuscany Region for the Medical Research and Public Health, Massa, Italy

Giuseppe Tarantini. University of Padova, Padova, Italy

- ITACTA - Italian Association of Cardiothoracic Anesthesia

Massimo Baiocchi. Policlinico Sant'Orsola, Bologna, Italy

Marco Ranucci. IRCCS Policlinico S. Donato, S. Donato Milanese, Milan, Italy

*Institutional collaborations*

- National

Domenico Mantoan. Agenzia Nazionale per i Servizi Sanitari Regionali (Age.Na.S), Rome, Italy

- Italian Regional Authorities

Rossana De Palma. Emilia Romagna Region

Salvatore Scondotto. Sicily Region

Anna Orlando. Piemonte Region

**Participating centers**

1. A.O.U. Città della Salute e della Scienza di Torino (TO) - Mauro Rinaldi, Stefano Salizzoni
2. A.O. S. Croce e Carle (CN) - Giuseppe Musumeci, Giorgio Baralis
3. A.O. SS. Antonio e Biagio e Cesare Arrigo (AL) - Gianfranco Pistis, Maurizio Reale
4. I.R.C.C.S Policlinico San Donato (San Donato Milanese - MI) - Francesco Bedogni, Giovanni Bianchi
5. I.R.C.C.S Multimedica (Sesto San Giovanni - MI) - Flavio Airoldi, Iassen Michev
6. Fondazione I.R.C.C.S. Policlinico San Matteo (PV) - Maurizio Ferrario, Umberto Canosi
7. ASST Lecco - Ospedale "A. Manzoni" (LC) - Luigi Piatti, Gianluca Tiberti
8. ASST degli Spedali Civili - Presidio Ospedaliero di Brescia (BS) - Federica Ettori (retired), Salvatore Curello, Marianna Adamo
9. I.R.C.C.S Ospedale San Raffaele (MI) - Antonio Colombo, Matteo Montorfano, Marco Ancona,
10. ASST Monza & Brianza - Ospedale S. Gerardo (MB) - Virgilio Colombo, Ivan Calchera
11. Fondazione Poliambulanza (BS) - Ornella Leonzi, Diego Maffeo
12. ASST Papa Giovanni XXIII (BG) - Orazio Valsecchi, Federica Roncali, Angelina Vassileva
13. Policlinico di Monza (MB) - Filippo Scalise, Giovanni Sorropago
14. A.O. di Padova - Centro Gallucci (PD) - Giuseppe Tarantini, Alessandro Schiavo
15. Hesperia Hospital (MO) - Giuseppe D'Anniballe, Davide Gabbieri
16. A.O.U. di Parma (PR) - Luigi Vignali, Michela Bollettino
17. A.O.U. Careggi (FI) - Carlo Di Mario, Francesco Meucci
18. A.O.U. Senese - Ospedale Santa Maria alle Scotte (SI) - Carlo Pierli (retired), Massimo Fineschi, Alessandro Iadanza
19. Fondazione Toscana Gabriele Monasterio - Ospedale del Cuore "G. Pasquinucci" (MS) - Sergio Berti, Giuseppa Lo Surdo
20. Ospedale San Filippo Neri (RM) - Giulio Speciale, Andrea Bisciglia
21. Fondazione Policlinico Universitario Agostino Gemelli IRCCS - Università Cattolica del Sacro Cuore (RM) - Carlo Trani, Diana Verdirosi
22. A.O. San Camillo Forlanini (RM) - Roberto Violini, Laura Zappavigna
23. A.O. San Giuseppe Moscati (AV) - Emilio Di Lorenzo, Michele Capasso
24. A.O.U. Federico II (NA)- Giovanni Esposito, Fabio Magliulo
25. A.O.U. OO.RR. San Giovanni di Dio e Ruggi d'Aragona (SA) - Pietro Giudice, Tiziana Attisano
26. A.O.U.C. Policlinico di Bari (BA) - Alessandro Santo Bortone, Emanuela De Cillis
27. A.O.U. Policlinico-Vittorio Emanuele, Università di Catania (CT) - Corrado Tamburino, Marco Barbanti
28. Centro Cuore Morgagni - Pedara (CT) - Sebastiano Immè, Martina Patanè

**OBSERVANT I RESEARCH GROUP**

The **Research Group and participating centers of the OBSERVANT I study** have been previously listed in: Barbanti M, Tamburino C, D'Errigo P, et al. Five-year outcomes of transfemoral transcatheter aortic valve replacement or surgical aortic valve replacement in a real world population. Circ Cardiovasc Interv. 2019;12:e007825 - Supplemental Material.
